# Beyond DNA Damage: The Impact of Tobacco Smoke and Its Derivatives on MicroRNAs in Lung Cancer Pathogenesis

**DOI:** 10.3390/cancers18213516

**Published:** 2026-11-01

**Authors:** Alina M. Perevalova, Lyudmila F. Gulyaeva, Vladimir O. Pustylnyak

**Affiliations:** 1 Institute for the Medicine and Medical Technology, Novosibirsk State University, Pirogova Street, 1, Novosibirsk 630090, Russia; a.perw@yandex.ru (A.M.P.); gulyaeva@niimbb.ru (L.F.G.); 2 Federal Research Center of Fundamental and Translational Medicine, Timakova Street, 2/12, Novosibirsk 630117, Russia

**Keywords:** lung cancer, tobacco smoking, microRNA

## Abstract

Tobacco smoking continues to represent the leading risk factor for multiple malignancies, most notably lung cancer (LC), which exhibits a complex molecular pathogenesis. While classical models prioritize direct DNA damage and mutagenesis, growing evidence points to the role of mechanisms not directly dependent on structural DNA lesions. In particular, dysregulation of oncogenic and tumor-suppressive microRNAs (miRNAs) has emerged as a promising area of research. Although numerous miRNAs potentially involved in LC pathogenesis have been reported to be altered by tobacco exposure, the available evidence is fragmented. This heterogeneity, compounded by the chemical complexity of tobacco smoke, prevents the formation of a comprehensive landscape of its pathogenetic mechanisms. This review aims to systematize existing data on tobacco smoke–induced changes in miRNA expression profiles in LC, with reference to individual tobacco smoke constituents, and to identify key biological processes likely modulated by these changes.

## Simple Summary

Lung cancer is strongly associated with tobacco smoking and involves multifaceted molecular changes. In addition to direct DNA damage, tobacco smoke interferes with microRNA networks. Although many miRNAs are reported to be altered in smoke-exposed lung cells, the evidence is fragmented. The chemical complexity of tobacco smoke further complicates interpretation. In this review, we aim to structure available evidence on miRNA alterations driven by tobacco smoke in lung cancer, considering individual tobacco smoke constituents.

## 1. Introduction

Lung cancer (LC) is one of the most common malignant neoplasms and remains the leading cause of cancer-related mortality [1]. Incidence and mortality trends vary across countries and are largely driven by differences in smoking prevalence [2]. The spectrum of molecular alterations involved in LC pathogenesis is heterogeneous and includes genetic, epigenetic, and post-transcriptional mechanisms, with molecular characteristics varying between different histological subtypes [3]. Non-small cell lung cancer (NSCLC) is the most common subtype and is characterized by high incidence and mortality rates. Despite available treatment options, including surgery, radiotherapy, chemotherapy, and targeted therapy, the 5-year survival rate remains comparatively low [4]. In this regard, the development of new drugs and corresponding targets, improvement of approaches to LC screening and the search for novel biomarkers remain highly important [2,4]. Tobacco smoking and the use of various electronic nicotine delivery systems are still considered the most significant risk factors for the development of lung cancer [2]. The search for potential diagnostic or prognostic biomarkers is often based on mechanisms associated with tobacco exposure [5,6]. Tobacco smoke has been shown to exert a range of biological effects, the best known of which is direct DNA damage. However, in recent years, increasing evidence has accumulated for carcinogenic mechanisms that are not directly related to its genotoxic effects. In particular, there is growing evidence that tobacco smoking can alter the expression levels of various tumor-suppressive and oncogenic microRNAs (miRNAs) under the influence of compounds present in tobacco smoke.

MicroRNAs are small, single-stranded non-coding RNA molecules known to regulate gene expression at the post-transcriptional level. Their influence has been associated with a wide range of human pathologies, including malignant tumors. Various microRNAs, both tumor-suppressive and oncogenic, are being investigated as prognostic markers or potential therapeutic targets because of their specificity and possible suitability for routine clinical use. However, changes in tissue or circulating microRNA levels can be influenced by a variety of factors, creating a complex network of molecular interactions. Under such conditions, identifying specific molecular markers associated with a particular pathogenic factor in a given group of pathologies may be challenging. The aim of this review is to systematize and summarize current data on the effects of tobacco smoke and its individual constituents on microRNA levels associated with the pathogenesis of lung cancer.

## 2. Derivatives of Tobacco Smoke and Their Carcinogenic Effects

It is well established that tobacco smoke contains numerous chemical compounds. With the improvement of modern analytical techniques, an increasing number of compounds have been identified in tobacco and tobacco smoke, currently exceeding 9500 [7]. Of these, 533 compounds are currently recognized as confirmed or possible human carcinogens [8]. In general, tobacco smoke components are divided into hydrocarbons, oxygen-containing components, nitrogen-containing components, and others. It has also been proposed to subdivide them into more specific groups according to their chemical class -hydrocarbons, amines, N-nitrosamines, esters, aldehydes, halogenated derivatives, nitrogenous compounds, phenolic compounds, inorganic compounds, and others [7].

There is some evidence of target tissue specificity for certain individual compounds. For example, with respect to the risk of developing lung cancer, polycyclic aromatic hydrocarbons (PAHs) and the tobacco-specific nitrosamine NNK (4-(methylnitrosamino)-1-(3-pyridyl)-1-butanone) are believed to have the most significant influence [9]. In addition, the influence of substances such as isoprene, ethylene oxide, ethyl carbamate, benzene, and various metals is suspected [10]. Numerous studies have demonstrated an association between exposure to specific compounds present in tobacco and tobacco smoke and the development of certain tumor subtypes [11,12,13,14,15].

Next, we will consider the main groups of tobacco smoke constituents and the molecular processes associated with carcinogenesis that have been described for them.

### 2.1. Polycyclic Aromatic Hydrocarbons (PAHs)

PAHs found in tobacco smoke include a wide variety of compounds; according to some data, their number reaches 510 [16]. Of these, 16 are classified as human carcinogens by IARC. The best known and most frequently encountered among these compounds are naphthalene, benz(a)anthracene, benzo(a)pyrene (BaP), and chrysene [7,17].

Due to their lipophilic nature, PAHs are able to freely diffuse through cell membranes. During subsequent metabolism, several molecular pathways involving different enzymes are activated, the best known of which is the CYP1A1/CYP1B1 and epoxide hydrolase pathway. Many PAHs are not directly carcinogenic and require metabolic activation, although carcinogenic potential may vary among individual PAHs. For BaP, CYP1A1/1B1 and epoxide hydrolase can either bioactivate or detoxify depending on context; activation begins with 7,8-epoxide formation, from which 7,8-diol is subsequently produced by epoxide hydrolase; in the presence of CYP1A1/1B1, this then forms the final carcinogenic product, 7,8-diol-9,10-epoxide (BPDE). BPDE subsequently reacts with the N2 position of guanosine in DNA, forming adducts [18]. It is believed that the formation of unstable adducts and impaired repair mechanisms lead to spontaneous depurination and the formation of apurinic DNA sites [19]. It is noted that DNA adduct formation is often a sequence-specific event, for example in genes such as TP53 and KRAS [18].

Another important product of PAH metabolism is reactive oxygen species (ROS). Their formation leads to disruption of the cellular redox balance, the development of oxidative stress, and the dysfunction of various macromolecules—DNA, RNA, proteins, and lipids [18].

One of the principal mechanisms of PAHs directly associated with carcinogenesis is the formation of a PAH complex with the aryl hydrocarbon receptor (AHR). After binding, the complex translocates to the nucleus, associates with the aryl hydrocarbon receptor nuclear translocator (ARNT), and interacts with xenobiotic response elements (XREs) in DNA. This binding leads, among other things, to induced CYP1A1/1A2/1B1 transcription and enhanced PAH metabolism to DNA-reactive metabolites. However, the roles of individual CYPs may vary and depend on context: while hepatic and pulmonary CYP1A1/1B1 metabolize PAHs to DNA-reactive metabolites, hepatic CYP1A2 may protect against lung tumor development by suppressing both liver and lung CYP1A1 enzymes, although the precise mechanisms remain incompletely understood. Inhibitors and inducers of these enzyme systems are proposed as potential therapeutic options [18].

Several epigenetic mechanisms associated with PAH exposure are also known. For example, BPDE has been shown to enhance the transcription of DNA methyltransferase and cadherin 13 genes [20]. Also, exposure of bronchial epithelial cells to BaP was found to induce hypermethylation of the *CDKN2A* (p16) gene, thereby preventing cell-cycle arrest and apoptosis. In the same study, higher levels of exposure biomarker (urinary 1-OHP) was demonstrated in peripheral lymphocytes of PAH-exposed workers [21]. PAH exposure has also been associated with histone modification. One of the proposed mechanisms is oxidative stress-induced covalent modification of HDAC2, leading to its reduced enzymatic activity [22,23]. An association has also been found between BaP exposure, suppression of DOT1 methyltransferase gene expression, decreased H3K79 methylation, and increased cellular sensitivity to gamma radiation [18].

### 2.2. Tobacco-Specific Nitrosamines (TSNAs)

Of this group of compounds, the tobacco-specific nitrosamines N-nitrosonicotine (NNN), nitrosamine ketone (NNK), N’-nitrosoanabasine (NAB), and N’-nitrosoanatabine (NAT) are found in tobacco smoke at the highest concentrations. Nicotine derivatives NNN and NNK possess the most pronounced carcinogenic properties [7]. These compounds are also procarcinogens and undergo metabolic activation, resulting in the formation of final carcinogenic products. NNN, for example, is metabolized through reactions such as N-oxidation of pyridine, hydroxylation of the pyrrolidine ring, and the formation of norcotinine, which also leads to the formation of products capable of interacting with DNA [24]. Moreover, the loss of DNA repair systems is noted to be highly significant for the development of the further carcinogenic potential of the resulting adducts. Each type of adduct exhibits a certain correlation with a preferred repair mechanism—for example, the BER, NER, and DNA alkyltransferase AGT (MGMT) systems are considered highly important [24]. Overall, it is noted that the formation of DNA adducts is an important initiating event in carcinogenesis, but not the only relevant process. The initiation and progression of tumor growth involves a cascade of processes both within tumor cells and in their microenvironment.

Nicotine and its derivatives are known to bind to nicotinic acetylcholine receptors (nAChRs). nAChRs are pentameric ligand-gated ion channels that can assemble as homomeric or heteromeric receptors, including α7 homopentamers and α4β2 heteropentamers. Nicotine itself binds preferentially to α4β2 heteropentamers. In smokers, the biological functions of α7 nAChRs are increased, whereas the functions of α4β2 nAChRs are impaired. Given the predominantly stimulatory role of α7 nAChRs and the inhibitory role of α4β2 nAChRs in tumor growth, this smoking-associated functional shift may create an environment favorable for the development and progression of malignant transformation. NNN, in turn, binds to αβ heteromers, whereas NNK binds to α7 homomers. Activation of these receptors, which are ligand-gated ion channels, results in membrane depolarisation and activation of protein kinase C, RAF1, ERK1, and ERK2 kinases, as well as the transcription factors FOS, JUN, and MYC, ultimately promoting cell proliferation. The presence of COPD in smokers may further enhance these carcinogenic effects through several mechanisms, including changes in the abundance and sensitivity of α7 nAChRs, reduced intracellular cAMP levels, and activation of RAF1-mediated cellular signaling. In NSCLC, exposure to NNK has been associated with activation of the PI3K/AKT signaling pathway and the transcription factor NF-κB. This, in turn, resulted in increased cell proliferation and inhibition of chemotherapy-induced apoptosis. In bronchial epithelial cell cultures, NNK-dependent activation of the ERK1/2, STAT1, NF-κB, and GATA3 signaling pathways has been observed, together with NNN-dependent activation of GATA3 and STAT1. NNK also acts as an agonist of β-adrenergic receptors (β-AdrRs). Activation of these receptors has been shown to induce cAMP-dependent transactivation of the epidermal growth factor receptor (EGFR), thereby increasing the malignant potential of cells [24].

It is believed that the direct genotoxic effect of TSNAs is insufficient to form a fully developed malignant phenotype in bronchial epithelial cells. Other processes, particularly those induced by the activation of intracellular signaling pathways, are also essential for the further malignant transformation of cells and the progression of tumor growth.

### 2.3. Aldehydes

Aldehydes are considered another significant group of tobacco smoke derivatives. Among these, formaldehyde, acetaldehyde, and crotonaldehyde are particularly prominent. In addition to tobacco smoke, these compounds may enter the body through other routes, including the consumption of fried foods, exposure to second-hand smoke, and inhalation of products generated by the incomplete combustion of fuel. They may also be produced endogenously during lipid peroxidation and the metabolism of polyamines and threonine [7]. Aldehydes can also form stable and unstable adducts with DNA, proteins, and lipids [16]. Moreover, each aldehyde appears to affect processes involved in the formation of DNA single-strand breaks, DNA–protein cross-links, and the functioning of DNA-repair systems in a distinct manner [25].

A considerable number of other components are also present in tobacco smoke. These include several volatile hydrocarbons, various structurally diverse groups of compounds, and certain heavy-metal compounds. Many of these substances are classified as Group 1 carcinogens; however, their specific molecular mechanisms remain to be clarified [7].

## 3. The Effect of Tobacco Smoke on microRNAs Associated with Lung Carcinogenesis

The literature contains a significant amount of evidence showing that tobacco smoke and its individual derivatives alter the levels of various microRNAs. However, both the volume of available information and the fragmented nature of the evidence do not currently allow us to form a definitive picture of their significance for carcinogenesis. The interpretation of existing data in both experimental studies and existing reviews is sometimes constrained by a lack of differentiation between diverse cellular and tissue contexts and the potentially divergent effects of various compound classes. Differences in the cellular and molecular context—and, in some cases, in the duration of exposure to particular factors—can play an important role in assessing the significance of specific molecular markers. Below, we aim to provide a more structured overview of microRNAs associated with the effects of tobacco smoke on lung cancer cells, based on their potential functional significance. For this review, we searched PubMed, Google Scholar, Web of Science, and Scopus databases for articles published up to 2025, using combinations of terms for tobacco smoke or its constituents (e.g., CSC, BaP, BPDE, NNK, nicotine, aldehydes, metals), microRNAs (e.g., miRNA, miR-) and lung cancer (e.g., NSCLC, SCLC, bronchial epithelial cells). Studies were eligible if they reported original miRNA data in human lung cells/tissues or in vivo models after such exposure. Reviews, abstracts and records without original miRNA data were excluded. Exploratory studies based on profiling, expression association or target prediction were retained. The level of target validation and the evidence category of each study are indicated in Table 1.

**Figure 1:**
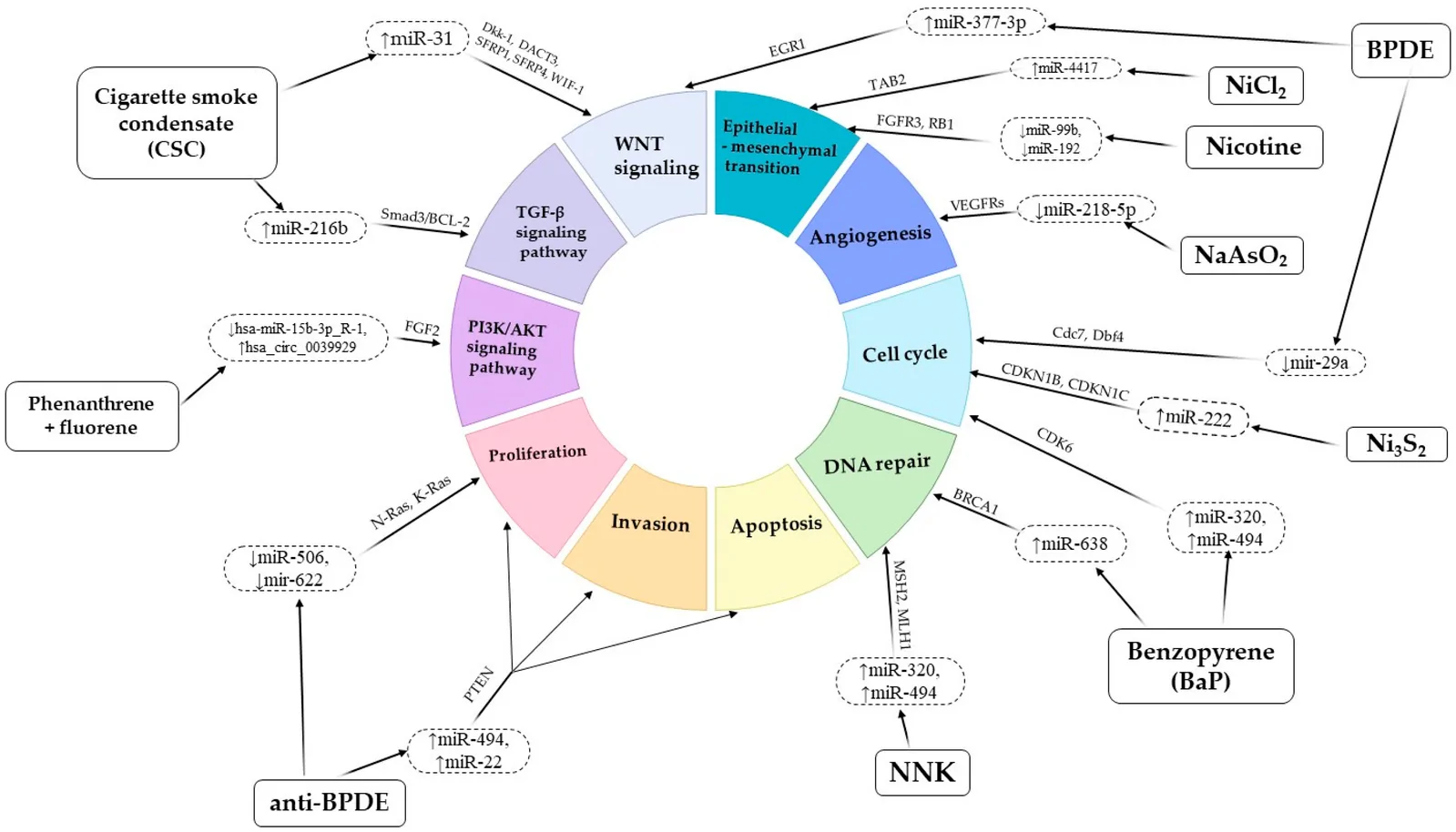
The complex landscape of tobacco smoke-induced microRNA alterations and their role in key oncogenic pathways in lung cancer. The data are presented schematically. Detailed information on specific changes in microRNA levels and associated cellular processes is provided in Table 1.

Examples of the effects of tobacco smoke and its components on changes in microRNA levels and the biological processes regulated by them are shown in Figure 1.

Exposure of lung cancer cell cultures to *tobacco smoke condensate* resulted in changes in the levels of 259 different microRNAs; for 39 of these, corresponding changes were identified in the levels of the proteins they are believed to regulate [26]. In a study investigating the effects of tobacco smoke condensate on gene-expression profiles during the malignant transformation of bronchial epithelial cells, changes were identified in the levels of 42 microRNAs and 753 mRNAs. Of these, as revealed by analysis of TCGA data, miR-96-5p, miR-93-5p, miR-106-5p, miR-190a-5p, miR-195-5p and miR-1-3p were observationally associated with a history of smoking. The same study noted some differences in the changes in microRNA levels between adenocarcinoma and squamous cell lung cancer. However, these data are correlational and require experimental validation [27]. Tobacco smoke condensate was shown to increase miR-31 levels in normal bronchial epithelial cells and lung cancer cells. This was accompanied by reduced expression of its target genes, DKK1 and DACT3, which act as antagonists of the WNT signaling pathway [28]. Similarly, tobacco smoke condensate increased miR-216b expression through acetylation of histones H3 and H4 near its genomic locus. This resulted in reduced levels of its target, SMAD3, and decreased activity of the TGF-β signaling pathway. These changes were also accompanied by increased BCL2 expression, which may contribute to chemoresistance in NSCLC cells by suppressing apoptosis [29]. Tobacco smoke condensate was also shown to reduce the level of miR-487b through DNA methylation. This effect was reportedly accompanied by attenuation of its inhibitory influence on SUZ12, BMI1, WNT5A, MYC, and KRAS, together with activation of the WNT signaling pathway [30].

There is also evidence of the influence of individual tobacco smoke derivatives on changes in the levels of microRNAs and their molecular targets in lung cancer cells. Most of the available data are focused on the effects of various *PAHs*. For example, treatment of bronchial epithelial cell cultures with BaP resulted in an increase in miR-638 levels, which is presumably associated with the suppression of BRCA1 and the development of DNA damage [31]. BaP exposure has also been associated with reduced levels of miR-152-3p, miR-142-5p, and miR-211-5p, accompanied by increased ALCAM levels and enhanced cell growth and proliferation [32]. Similarly, exposure to BaP and its active form BPDE was associated with increased miR-377-3p levels and reduced EGR1 levels; EGR1 is believed to be involved in the activity of the WNT signaling pathway [33]. In lung cancer cell cultures treated with BPDE alone, reduced miR-29a levels and increased levels of its target, the cyclin-dependent kinase Cdc7, were observed [34]. Other studies have associated exposure to the BPDE stereoisomer (anti-BPDE) with increased levels of miR-494 and miR-22, the potential target of which is the tumor suppressor protein PTEN [35,36]. A decrease in miR-506 and miR-622 levels, which potentially target N-Ras and K-Ras mRNAs, respectively, was demonstrated in anti-BPDE-transformed 16HBE-T cells [37,38]. Anti-BPDE has likewise been reported to reduce the level of miR-542-3p and to enhance cell proliferation in lung cancer cell cultures [39]. Exposure to anti-BPDE was also associated with reduced miR-10a levels and increased levels of its target, HOXA1 [40]. Increased levels of miR-320 and miR-494 were detected following treatment with BaP; this was accompanied by reduced CDK6 expression and disruption of the G1/S transition [41]. Treatment of lung cancer cells with other PAH derivatives, phenanthrene and fluorene, was associated with reduced levels of hsa-miR-15b-3p_R-1, accompanied by activation of the PI3K/AKT signaling pathway and enhancement of the epithelial–mesenchymal transition [42].

Evidence is also available regarding the effects of other tobacco smoke constituents on microRNA levels. *Nicotine* alters the expression of multiple microRNAs in lung and other cell types, as summarized in [43]. For example, the nicotine-induced downregulation of miR-99b and miR-192, with consequent upregulation of FGFR3 and RB1, has been demonstrated in NSCLC cells. These changes were accompanied by enhanced cell proliferation and epithelial–mesenchymal transition [44]. A broader overview of nicotine-responsive miRNAs and their nAChR-linked signaling is provided in [43]. After exposure to NNK, a decrease in the levels of miR-101, miR-126-5p, miR-199a and miR-34b in lung tissue in rats was demonstrated. CYP2A3, which participates in NNK metabolism, was proposed as a potential target of miR-126-5p. This may represent a mechanism capable of enhancing the carcinogenic properties of NNK [45]. In laboratory animals exposed to NNK, increased levels of miR-144-3p, miR-29c-3p, and miR-20a-5p, together with reduced levels of miR-328-3p and let-7d-3p, were observed in transformed lung tissue. A decrease in miR-328-3p levels was associated with the suppressive effect of the long non-coding RNA XIST [46]. In lung squamous cell carcinoma cells treated with NNK, increased levels of miR-21 and miR-155 and reduced levels of miR-422a were observed. These changes were accompanied by suppression of MSH2 and MLH1, which participate in the mismatch repair (MMR) system [47].

The effects of another group of tobacco smoke derivatives—*aldehydes*—on microRNA levels have also been investigated. Exposure of lung adenocarcinoma cell cultures to formaldehyde resulted in reduced levels of 89 different microRNAs. The most pronounced changes were observed in miR-33, miR-330, miR-181a, and miR-10b levels; subsequent bioinformatic analysis indicated the possible involvement of the NF-κB and IL-8 signaling pathways [48]. When studying the effects of propanaldehyde, butanaldehyde, and pentanaldehyde on the same cell cultures, changes were found in the levels of 15, 25, and 10 different microRNAs, respectively, with only miR-21 found to be common to these groups. The IL-6 and IL-8 signaling pathways were proposed as potential targets for all three groups [49]. Exposure of human pulmonary fibroblast cultures to different concentrations of acrolein resulted in changes in the levels of more than 30 microRNAs. Bioinformatic predictions suggest a possible role for extracellular matrix protein interactions in mediating the effects of these microRNAs [50].

Changes in microRNA levels have also been observed following exposure to *heavy metal compounds*. For example, arsenic exposure has been associated with reduced miR-200b levels, epithelial–mesenchymal transition, and the development of a malignant cellular phenotype [51]. Treatment of bronchial epithelial cells with an arsenic compound resulted in reduced levels of miR-218-5p and increased levels of its target, EGFR. The proposed mechanisms included the regulation of cell proliferation, growth, migration, and angiogenesis [52]. Exposure to beryllium sulphate resulted in increased levels of 88 microRNAs and reduced levels of 91 microRNAs in bronchial epithelial cells; the principal molecular pathways implicated were the MAPK and VEGF pathways [53]. Similarly, treatment with cadmium chloride altered the levels of 8 microRNAs. Subsequent gene network analysis showed that most of the target genes were regulated by miR-27b-3p. The proposed biological processes involved included the cellular response to stress, regulation of transcription and the cell cycle, the p53 signaling pathway, and several others [54]. Exposure to nickel chloride was associated with increased miR-4417 levels and reduced expression of its target gene, TAB2. These changes may promote carcinogenic processes by affecting fibronectin expression and the epithelial–mesenchymal transition [55]. Nickel sulphide exposure has also been associated with increased miR-222 levels, which may stimulate cell proliferation both in vitro and in vivo through effects on its targets, CDKN1B and CDKN1C [56].

Some data are also available on the effects of electronic nicotine delivery systems on cellular microRNA levels. In one study, bronchial epithelial cell cultures were treated with both nicotine-containing and nicotine-free vaping liquids, as well as condensates generated from their aerosols. Significant differences in the levels of several microRNAs were detected compared with both the untreated control group and the group of cells treated with tobacco smoke condensate. Significant increases were confirmed in the levels of miR-26a-2-3p, miR-126-5p, miR-140-5p, miR-29a-5p, miR-374a-3p, and miR-147b, with the most pronounced increase observed for miR-126-5p. For miR-126-5p, reduced levels of its targets MYC and MRGPRX3 were also demonstrated, which was not observed in the group of cells treated with tobacco smoke condensate [57]. It is therefore reasonable to assume that differences in microRNA-expression profiles following exposure to tobacco smoke and e-cigarette condensates may be explained by differences in the composition and concentrations of the inhaled constituents.

**Figure 2:**
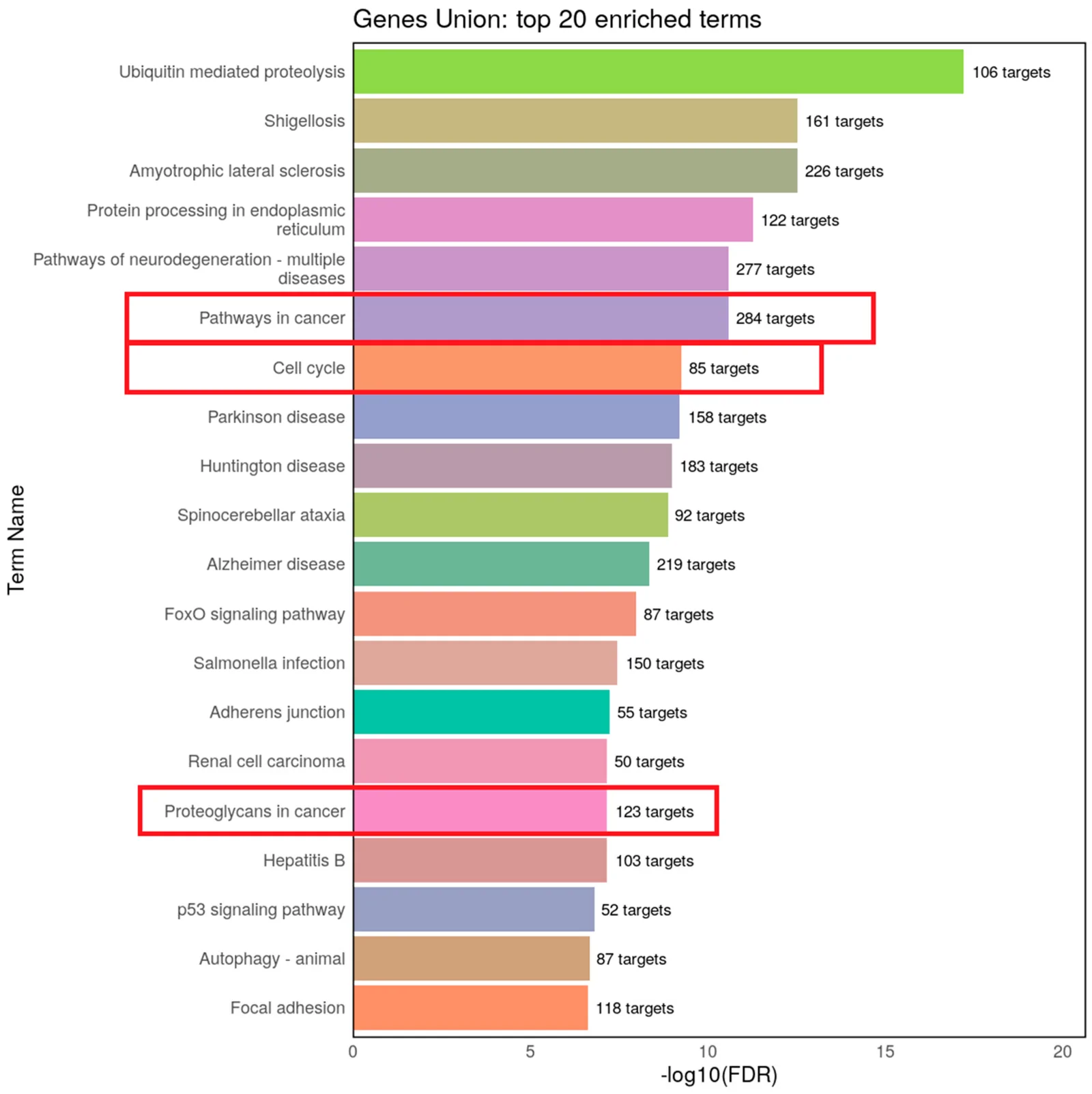
KEGG pathway enrichment analysis of the predicted target genes for the experimentally supported miRNAs listed in Table 1 [58]. Target genes were predicted with DIANA-miRPath v4.0 using default settings, and pathways with FDR < 0.05 were considered significantly enriched. Framed pathways indicate carcinogenesis-related processes. Results are based on bioinformatic prediction and require experimental validation.

The changes in microRNA levels and their targets described above, together with the putative biological processes involved, are summarized in Table 1. To perform a preliminary analysis of the potential molecular pathways influenced by the listed miRNAs, a bioinformatic KEGG pathway enrichment analysis was performed using DIANA-miRPath v4.0 database (Figure 2) [58]. As shown by the analysis, a significant portion of the identified molecular pathways and several hundred predicted targets are associated with processes involved in carcinogenesis.

**Table 1:** Effects of tobacco smoke constituents on microRNA levels, their targets, and the putative biological processes involved in lung cancer cells. Abbreviations: D—direct miRNA–target interaction experimentally validated, T—target expression/protein level changed, but direct binding not demonstrated, H—human tissue-relevant associations demonstrated, ↑/↓—increased/decreased expression level.

Substance(s)	Experimental Model	Exposure Dose and Duration	Methods	MicroRNA	Upstream Regulators	Downstream Targets	Processes	References
	Tobacco-relevant exposure evidence							
Cigarette smoke condensate (CSC)	Normal human cell lines (SAEC, NHBE, HBEC), human NSCLC and SCLC cell lines (Calu-6, H841, H1299, H1650, and H1975), Human tissues (lung cancer/normal)	CSC 1%; 5 days	Luciferase reporter assay, proliferation assay, qRT-PCR, ChIP, Western blotting, MicroRNA microarray, Ago CLIP, Murine xenograft experiments	↑ miR-31 (H)	C/EBP-β, H3K4Me3, H3K9/14Ac	Dkk-1 (D), DACT3 (D), SFRP1 (T), SFRP4 (D), WIF-1 (T)	WNT signaling pathway	[28]
	Human NSCLC cell lines (A549, NCI-H460), Normal human cell lines (Beas2B, HPL1A)	CSC 0–200 µg/mL; 6 days	Immunoblotting, immunoprecipitation, luciferase reporter assay, qRT-PCR, ChIP	↑ miR-216b	Histones H3, H4 acetylation	Smad3 (D), BCL-2 (T)	TGF-β signaling pathway, apoptosis, chemoresistance	[29]
	Normal human cell lines (SAEC, HBEC), human NSCLC and SCLC cell lines (Calu-6, H841), Human tissues (lung cancer/normal)	CSC (0.025 mg/mL); 5 days	Luciferase reporter assay, proliferation assay, qRT-PCR, Ago CLIP, MeDIP assay, bisulfite sequencing, MNase protection analysis, in vitro invasion, cell cycle, senescence, and apoptosis assays, murine xenograft experiments	↓ miR-487b (H)	CpG methylation, de novo nucleosome occupancy, H2AZ↓, TCF1↓	SUZ12 (D), BMI1 (D), WNT5A (D), MYC (D), KRAS (D)	WNT signaling pathway, proliferation, invasion	[30]
	Constituent-level mechanistic evidence: PAHs							
Benzopyrene (BaP)	Normal human cell lines (16HBE14o-, HBE), Human tissues (NSCLC/normal)	BaP (5 μM, 10 μM, 20 μM, 40 μM); 24 h	luciferase reporter assay, miRNA array, CBMN assay, alkaline comet assay qRT-PCR, immunoblotting	↑ miR-638 (H)	Not reported	BRCA1 (T)	DNA repair	[31]
	Normal human cell line (HBE), BaP-transformed HBE cell line (THBEc1)	Not reported	miRNA sequencing, RT-PCR, MiRNA mimics transfection, Western blotting, CRISPR/Cas9, immunohistochemistry, colony formation, transwell migration assays, nude mouse tumorigenesis assay	↓ miR-152-3p, ↓ miR-142-5p, ↓ miR-211-5p	Not reported	ALCAM (T)	Proliferation, growth	[32]
	Murine bronchial epithelial cells	BaP (0.01 µM, 0.1 µM, 1 µM); 48 h	qRT-PCR, cell cycle analysis, flow cytometry	↑miR-320, ↑miR-494	Not reported	CDK6 (T)	Cell cycle	[41]
BPDE	Normal human cell line (BEAS-2B), 293T cell line, Murine tissue samples	BPDE (0.2 µM, 0.5 µM); for 2 h once a week for 12 weeks	qRT-PCR, immunoblotting, animal models, soft agar assay, scratch test, transwell assay, immunohistochemistry, luciferase reporter assay, immunofluorescence	↑ miR-377-3p	Not reported	EGR1 (D)	WNT signaling pathway	[33]
	Pulmonary fibroblast cell line (MRC-5), Human NSCLC cell lines (A549, H1299)	BPDE (100 nM, 600 nM); 8 h	qRT-PCR, Western blotting, luciferase reporter assay, colony formation assay, flow cytometry	↓ miR-29a	Not reported	CDC7 (D), DBF4 (D)	Cell cycle	[34]
anti-BPDE	Normal human cell line (16HBE-N), anti-BPDE transformed cell lines (16HBE-T)	Not reported	qRT-PCR, Western blotting, luciferase reporter assay, apoptosis assay, scratch test, soft agar assay	↑ miR-494, ↑ miR-22	Not reported	PTEN (D)	Proliferation, growth, motility, apoptosis	[35,36]
	Normal human cell line (16HBE-N), anti-BPDE transformed cell lines (16HBE-T)	Not reported	qRT-PCR, Western blotting, cell viability assay, cell cycle analysis, apoptosis assay, soft agar assay, murine xenograft experiments, luciferase reporter assay	↓ miR-506, ↓ miR-622	Not reported	NRAS (T), KRAS (D)	Proliferation, cell cycle, growth	[37,38]
	Normal human cell line (16HBE-N), anti-BPDE transformed cell lines (16HBE-T)	Not reported	qRT-PCR, miRNA mimic transfection, proliferation, cell cycle, apoptosis assays, soft agar assay	↓ miR-542-3p	Not reported	Not reported	Proliferation, cell cycle, colony formation	[39]
	Normal human cell line (16HBE-N), anti-BPDE transformed cell lines (16HBE-T)	Not reported	MiRNA microarray, qRT-PCR, bioinformatics analysis	↓ miR-10a	Not reported	HOXA1 (T)	p44/42 MAP kinase pathway	[40]
Phenanthrene (phe), fluorene (flu) (mixture)	Human NSCLC cell line (A549)	Phe + Flu (mixture, 600 μmol/L); 24 h	RNA sequencing, bioinformatics analysis, transfection assay, qRT-PCR, Western blotting	↓ miR-15b-3p_R-1	hsa_circ_0039929	FGF2 (T)	PI3K/AKT signaling pathway, epithelial–mesenchymal transition	[42]
	Constituent-level mechanistic evidence: nicotine and TSNAs							
Nicotine	Human NSCLC cell lines (A549, NCI-H460)	Nicotine (100 μM); 48 h	qRT-PCR, cell transfection, MTT assay, Western blotting, luciferase reporter assay	↓ miR-99b, ↓ miR-192	Not reported	FGFR3 (D), RB1 (D)	Proliferation, epithelial–mesenchymal transition	[44]
NNK	Male F344 rats (lung tissues)	10 p.p.m. of NNK in the drinking water	miRNA microarray, Northern blotting, RT-PCR, luciferase reporter assay, Western blotting	↓ miR-126-5p	Not reported	CYP2A3 (D)	NNK metabolism	[45]
	(1) A/J mice, (2) Murine normal and lung cancer cell lines (MLE-12, LLC), Human normal and LUAD cell lines (BEAS-2B, A549)	(1) NNK (100 mg/kg) i.p. (2) NNK (0.1 mg/mL, 0.5 mg/mL); 48 h	qRT-PCR, RNA immunoprecipitation assay, luciferase reporter assay, MTT assay, flow cytometry, Western blotting, immunofluorescence, colony formation and migration ability assays	↓ miR-328-3p	lncRNA XIST	FZD7 (D)	Apoptosis, cell cycle, WNT signaling pathway	[46]
	Human hypopharyngeal and lung squamous cancer cell lines (HTB-43, NCI-H1703)	NNK (1 μM, 2 μM); 24 h	Immunocytochemistry, immunofluorescence, Western blotting, qRT-PCR, cell transfection, immunosorbent assay, cell viability assay	↑ miR-21, ↑ miR-155 ↓ miR-422a	Not reported	MSH2 (T), MLH1 (T)	DNA repair	[47]
	Constituent-level mechanistic evidence: aldehydes							
Formaldehyde	Human LUAD cell line (A549)	1 ppm formaldehyde; 4 h	Coupled enzymatic assay, miRNA microarray, qRT-PCR, enrichment and network analysis, immunosorbent assay	↓ miR-33, ↓ miR-330, ↓ miR-181a, ↓ miR-10b	Not reported	Not reported	NFκB and IL-8 signaling pathways	[48]
	Constituent-level mechanistic evidence: metals							
Sodium arsenite (NaAsO_2_)	Normal human cell lines (HBECs, NHBEs)	NaAsO_2_ (2.5 μM); overnight every 3 or 4 days for 16 weeks	Growth assay, murine xenograft experiments, miRNA microarray, luciferase reporter assay, DNA methylation analysis, RT-PCR, Western blotting, immunofluorescence	↓ miR-200b	DNA methylation	ZEB1 (D), ZEB2 (D)	Epithelial–mesenchymal transition	[51]
	Normal human cell line (BEAS-2B)	NaAsO_2_ (1 μM); 24 weeks	Immunoblotting, qRT-PCR, luciferase reporter assay, viral transduction, immunohistochemistry, cell proliferation, colony formation, wound healing and tube formation assays, nude mice experiments	↓ miR-218-5p	Not reported	EGFR (D), VEGFR1 (T), VEGFR2 (T), VEGFR3 (T)	Proliferation, migration, colony formation, angiogenesis	[52]
Beryllium sulfate (BeSO_4_)	Normal human cell line (16HBE)	BeSO_4_ (0–300 μmol/L); 48 h	Cell viability assay, ELISA, Western blotting, miRNA sequencing, qRT-PCR, bioinformatics analysis	↑ miR-504 ↑ miR-4485 ↓ miR-663b ↓ miR-4417, etc.	Not reported	Not reported	MAPK and VEGF signaling pathways	[53]
Cadmium chloride (CdCl_2_)	Normal human cell line (16HBE)	CdCl_2_ (5 μmol/L); 14 weeks	miRNA microarray, qRT-PCR, bioinformatics analysis	↑ miR-27b-3p	Not reported	Not reported	MAPK signaling pathway, cell cycle, transcription regulation, etc.	[54]
Nickel (II) chloride (NiCl_2_)	(1) Human normal and LUAD cell lines (BEAS-2B and A549) (2) BALB/AnN.Cg-Foxnlnu/CrlNarl nude mice	(1) NiCl_2_ (0.5–1 mM); 72 h (2) NiCl_2_ (0, 20 or 100 mg/kg/day) by oral gavage; 60 days	Western blotting, RNA microarray, qRT-PCR, cell transfection, luciferase reporter assay, murine xenograft experiments	↑ miR-4417	TGF-β signaling	TAB2 (D)	Epithelial–mesenchymal transition	[55]
Trinickel disulfide (Ni_3_S_2_)	(1) Wistar rats (2) Normal human cell line (16HBE)	(1) Ni_3_S_2_ (10 mg, i.m.) (2) Ni_3_S_2_ (0.6 μg/cm^2^); 24 h	Soft agar assay, qRT-PCR, murine xenograft experiments	↑ miR-222	Not reported	CDKN1B (T), CDKN1C (T)	Cell cycle	[56]

## 4. Conclusions

The literature contains substantial evidence that tobacco smoke and its individual constituents affect carcinogenic mechanisms operating at the post-transcriptional level. As noted, the influence of tobacco smoke components and their derivatives is not limited to their direct genotoxic effects, leading to direct DNA damage. Evidence indicates that exposure to these derivatives alters the expression levels of numerous microRNAs. However, the specific mechanisms responsible for these changes, as well as the molecular pathways affected by them, remain to be elucidated and experimentally validated. The molecular context appears to depend not only on the tissue and cellular origin of the tumor, but also on the amount and concentration of individual chemical compounds present in inhaled smoke. In addition to the extensive accumulated information on changes in cellular levels of microRNAs themselves, various molecular processes are also involved, including proliferation, cell cycle progression, DNA repair, transcriptional regulation, angiogenesis, and many others.

A crucial caveat in interpreting the presented data is the use of high, often supraphysiological, concentrations of tobacco smoke derivatives in in vitro experiments. This implies that not all described microRNA changes may be observed in the actual tissues of long-term smokers, underscoring the need for validation of these findings in clinical samples and further assessment of their diagnostic or prognostic value. Moreover, the observed differences in microRNA profiles cannot currently be considered strictly specific due to variations in experimental models and conditions. This limitation highlights the need for further research using standardized and directly comparable experimental designs to identify specific microRNA profiles and clarify their potential differences.

At present, it is not possible to determine whether specific microRNAs, or particular changes in their levels, are uniquely associated with individual tobacco smoke derivatives. Nevertheless, this issue represents a promising area for further research. The majority of literature data regarding alterations in microRNA levels focuses on the effects of PAH and nicotine derivatives; however, evidence exists concerning the impact of various other chemical compounds, highlighting the need for further research. The evidence reviewed here indicates that exposure to tobacco and its individual constituents alters the levels of at least several hundred microRNAs, whose pathogenetic significance remains to be established. Notably, the previous literature has already demonstrated differences in microRNA expression profiles among various histological subtypes of lung cancer, as well as within groups of lung adenocarcinoma patients depending on their smoking status [59,60]. At the same time, the apparent differences in molecular profiles induced by different compounds suggest that fundamental studies aimed at identifying specific pathogenetic interactions—and, ultimately, clinically relevant biomarkers—should also take into account the chemical composition of the substances used. It remains to be seen which tobacco derivatives are most important for tumor growth and to characterize the specific molecular profiles of changes that they induce in different cellular and tissue contexts. Such data could potentially help identify the most promising markers for prognostic assessment and reveal new therapeutic targets in smoking-related lung cancer.

## Data Availability

No new data were created or analyzed in this study. Data sharing is not applicable to this article.

## References

[1] Guo L., Zhu C., Cai L., Zhang X., Fang Y., Chen H., Yang H. (2024). Global burden of lung cancer in 2022 and projected burden in 2050. Chin. Med. J..

[2] Leiter A., Veluswamy R.R., Wisnivesky J.P. (2023). The global burden of lung cancer: Current status and future trends. Nat. Rev. Clin. Oncol..

[3] Smolarz B., Łukasiewicz H., Samulak D., Piekarska E., Kołaciński R., Romanowicz H. (2025). Lung Cancer—Epidemiology, Pathogenesis, Treatment and Molecular Aspect (Review of Literature). Int. J. Mol. Sci..

[4] Li Y., Yan B., He S. (2023). Advances and challenges in the treatment of lung cancer. Biomed. Pharmacother..

[5] Cha S.-R., Jang J., Park S.-M., Ryu S.M., Cho S.-J., Yang S.-R. (2023). Cigarette Smoke-Induced Respiratory Response: Insights into Cellular Processes and Biomarkers. Antioxidants.

[6] Bergman M., Fountoukidis G., Smith D., Ahlgren J., Lambe M., Valachis A. (2022). Effect of Smoking on Treatment Efficacy and Toxicity in Patients with Cancer: A Systematic Review and Meta-Analysis. Cancers.

[7] Li Y., Hecht S.S. (2022). Carcinogenic components of tobacco and tobacco smoke: A 2022 update. Food Chem. Toxicol..

[8] International Agency for Research on Cancer, Monographs. 2022 Available: IARC Monographs on the Identification of Car-cinogenic Hazards to Humans. https://monographs.iarc.who.int/monographs-available/.

[9] Hecht S.S., DeMarini D.M., Baan R.A., Stewart B.W., Straif K. (2019). Tobacco smoke and its constituents. Tumour Site Concordance and Mechanisms of Carcinogenesis.

[10] Hecht S.S., DeVita V.T., Lawrence T.S., Rosenberg S.A. (2008). Etiology of cancer: Tobacco. DeVita, Hellman, and Rosenberg’s Cancer: Principles and Practice of Oncology.

[11] Shen J., Sun N., Wang J., Zens P., Kunzke T., Buck A., Prade V.M., Wang Q., Feuchtinger A., Hu R. (2023). Patterns of Carbon-Bound Exogenous Compounds Impact Disease Pathophysiology in Lung Cancer Subtypes in Different Ways. ACS Nano.

[12] Stepanov I., Sebero E., Wang R., Gao Y.-T., Hecht S.S., Yuan J.-M. (2013). Tobacco-specific*N*-nitrosamine exposures and cancer risk in the Shanghai cohort study: Remarkable coherence with rat tumor sites. Int. J. Cancer.

[13] (2010). IARC Working Group on the Evaluation of Carcinogenic Risks to Humans. Some non-heterocyclic polycyclic aromatic hy-drocarbons and some related exposures. IARC Monogr. Eval. Carcinog. Risks Hum..

[14] Hecht S.S. (1998). Biochemistry, Biology, and Carcinogenicity of Tobacco-Specific *N*-Nitrosamines. Chem. Res. Toxicol..

[15] Balbo S., James-Yi S., Johnson C.S., O’sullivan M.G., Stepanov I., Wang M., Bandyopadhyay D., Kassie F., Carmella S., Upadhyaya P. (2013). (S)-N’-Nitrosonornicotine, a constituent of smokeless tobacco, is a powerful oral cavity carcinogen in rats. Carcinogenesis.

[16] Sapkota M., Wyatt T.A. (2015). Alcohol, Aldehydes, Adducts and Airways. Biomolecules.

[17] Jeffery J., Carradus M., Songin K., Pettit M., Pettit K., Wright C. (2018). Optimized method for determination of 16 FDA polycyclic aromatic hydrocarbons (PAHs) in mainstream cigarette smoke by gas chromatography–mass spectrometry. BMC Chem..

[18] Stading R., Gastelum G., Chu C., Jiang W., Moorthy B. (2021). Molecular mechanisms of pulmonary carcinogenesis by polycyclic aromatic hydrocarbons (PAHs): Implications for human lung cancer. Semin. Cancer Biol..

[19] Ewa B., Danuta M. (2016). Polycyclic aromatic hydrocarbons and PAH-related DNA adducts. J. Appl. Genet..

[20] Damiani L.A., Yingling C.M., Leng S., Romo P.E., Nakamura J., Belinsky S.A. (2008). Carcinogen-Induced Gene Promoter Hypermethylation Is Mediated by DNMT1 and Causal for Transformation of Immortalized Bronchial Epithelial Cells. Cancer Res..

[21] Yang P., Ma J., Zhang B., Duan H., He Z., Zeng J., Zeng X., Li D., Wang Q., Xiao Y. (2012). CpG Site–Specific Hypermethylation of *p16INK4α* in Peripheral Blood Lymphocytes of PAH-Exposed Workers. Cancer Epidemiol. Biomark. Prev..

[22] Marwick J.A., Kirkham P.A., Stevenson C.S., Danahay H., Giddings J., Butler K., Donaldson K., MacNee W., Rahman I. (2004). Cigarette Smoke Alters Chromatin Remodeling and Induces Proinflammatory Genes in Rat Lungs. Am. J. Respir. Cell Mol. Biol..

[23] Klingbeil E.C., Hew K.M., Nygaard U.C., Nadeau K.C. (2014). Polycyclic aromatic hydrocarbons, tobacco smoke, and epigenetic remodeling in asthma. Immunol. Res..

[24] Xue J., Yang S., Seng S. (2014). Mechanisms of Cancer Induction by Tobacco-Specific NNK and NNN. Cancers.

[25] Grafström R.C. (1990). In vitro studies of aldehyde effects related to human respiratory carcinogenesis. Mutat. Res. Genet. Toxicol..

[26] Advani J., Subbannayya Y., Patel K., Khan A.A., Patil A.H., Jain A.P., Solanki H.S., Radhakrishnan A., Pinto S.M., Sahasrabuddhe N.A. (2017). Long-Term Cigarette Smoke Exposure and Changes in MiRNA Expression and Proteome in Non-Small-Cell Lung Cancer. OMICS A J. Integr. Biol..

[27] Wang J., Yu X.-F., Ouyang N., Zhao S., Yao H., Guan X., Tong J., Chen T., Li J.-X. (2019). MicroRNA and mRNA Interaction Network Regulates the Malignant Transformation of Human Bronchial Epithelial Cells Induced by Cigarette Smoke. Front. Oncol..

[28] Xi S., Yang M., Tao Y., Xu H., Shan J., Inchauste S., Zhang M., Mercedes L., Hong J.A., Rao M. (2010). Cigarette Smoke Induces C/EBP-β-Mediated Activation of miR-31 in Normal Human Respiratory Epithelia and Lung Cancer Cells. PLoS ONE.

[29] Vu T., Yang S., Datta P.K. (2020). MiR-216b/Smad3/BCL-2 Axis Is Involved in Smoking-Mediated Drug Resistance in Non-Small Cell Lung Cancer. Cancers.

[30] Xi S., Xu H., Shan J., Tao Y., Hong J.A., Inchauste S., Zhang M., Kunst T.F., Mercedes L., Schrump D.S. (2013). Cigarette smoke mediates epigenetic repression of miR-487b during pulmonary carcinogenesis. J. Clin. Investig..

[31] Li D., Wang Q., Liu C., Duan H., Zeng X., Zhang B., Li X., Zhao J., Tang S., Li Z. (2011). Aberrant Expression of miR-638 Contributes to Benzo(a)pyrene-Induced Human Cell Transformation. Toxicol. Sci..

[32] Li L., Chen J., Wang Y., Zhou C., Ma X., Fu J., Yao B., Zhao P. (2020). MicroRNA expression profiling and the role of ALCAM modulating tumor growth and metastasis in benzo[a]pyrene-transformed 16HBE cells. Toxicology.

[33] Ke X., He L., Wang R., Shen J., Wang Z., Shen Y., Fan L., Shao J., Qi H. (2021). miR-377-3p-mediated EGR1 downregulation promotes B[a]P-induced lung tumorigenesis by Wnt/β-catenin transduction. Front. Oncol..

[34] Barkley L.R., Santocanale C. (2013). MicroRNA-29a regulates the benzo[a]pyrene dihydrodiol epoxide-induced DNA damage response through Cdc7 kinase in lung cancer cells. Oncogenesis.

[35] Liu L., Jiang Y., Zhang H., Greenlee A.R., Han Z. (2010). Overexpressed miR-494 down-regulates PTEN gene expression in cells transformed by anti-benzo(a)pyrene-trans-7,8-dihydrodiol-9,10-epoxide. Life Sci..

[36] Liu L., Jiang Y., Zhang H., Greenlee A.R., Yu R., Yang Q. (2010). miR-22 functions as a micro-oncogene in transformed human bronchial epithelial cells induced by anti-benzo[a]pyrene-7,8-diol-9,10-epoxide. Toxicol. Vitr..

[37] Zhao Y., Liu H., Li Y., Wu J., Greenlee A.R., Yang C., Jiang Y. (2011). The role of miR-506 in transformed 16HBE cells induced by anti-benzo[a]pyrene-trans-7,8-dihydrodiol-9,10-epoxide. Toxicol. Lett..

[38] Han Z., Yang Q., Liu B., Wu J., Li Y., Yang C., Jiang Y. (2011). MicroRNA-622 functions as a tumor suppressor by targeting K-Ras and enhancing the anticarcinogenic effect of resveratrol. Carcinogenesis.

[39] Zhao Y., Liu H.-Y., Li Y.-Q., Jiang Y.-G. (2011). [Effect of miR-542-3p on carcinogenesis induced by anti-benzo(a) pyrene-7,8-diol-9,10-epoxide]. Zhonghua Yu Fang Yi Xue Za Zhi.

[40] Shen Y.-L., Jiang Y.-G., Greenlee A.R., Zhou L.-L., Liu L.-H. (2009). MicroRNA Expression Profiles and MiR-10a Target in Anti-benzo[a] pyrene-7, 8-diol-9, 10-epoxide-transformed Human 16HBE Cells. Biomed. Environ. Sci..

[41] Duan H., Jiang Y., Zhang H., Wu Y. (2010). MiR-320 and miR-494 affect cell cycles of primary murine bronchial epithelial cells exposed to benzo[a]pyrene. Toxicol. Vitr..

[42] Huang Y., Huang Y., Wang H., Zhang H., Shi L., Li C., Li X., Zeng Y., Liu Y., Wu M. (2022). The effect of low molecular weight-polycyclic aromatic hydrocarbons responsive hsa_circ_0039929/hsa-miR-15b-3p_R-1/FGF2 circuit on inflammatory response of A549 cells via the PI3K/AKT pathway and epithelial-mesenchymal transition process. Environ. Toxicol..

[43] Hajiasgharzadeh K., Naghipour B., Shahabi P., Dastmalchi N., Alipour M.R. (2023). The role of microRNAs in nicotine signaling. EXCLI J..

[44] Du X., Qi F., Lu S., Li Y., Han W. (2018). Nicotine upregulates FGFR3 and RB1 expression and promotes non-small cell lung cancer cell proliferation and epithelial-to-mesenchymal transition via downregulation of miR-99b and miR-192. Biomed. Pharmacother..

[45] Kalscheuer S., Zhang X., Zeng Y., Upadhyaya P. (2008). Differential expression of microRNAs in early-stage neoplastic transformation in the lungs of F344 rats chronically treated with the tobacco carcinogen 4-(methylnitrosamino)-1-(3-pyridyl)-1-butanone. Carcinogenesis.

[46] Li B., Li X., Jiang Z., Zhou D., Feng Y., Chen G., Li N. (2024). LncRNA XIST modulates miR-328-3p ectopic expression in lung injury induced by tobacco-specific lung carcinogen NNK both in vitro and in vivo. Br. J. Pharmacol..

[47] Doukas S.G., Vageli D.P., Lazopoulos G., Spandidos D.A., Sasaki C.T., Tsatsakis A. (2020). The Effect of NNK, A Tobacco Smoke Carcinogen, on the miRNA and Mismatch DNA Repair Expression Profiles in Lung and Head and Neck Squamous Cancer Cells. Cells.

[48] Rager J.E., Smeester L., Jaspers I., Sexton K.G., Fry R.C. (2011). Epigenetic Changes Induced by Air Toxics: Formaldehyde Exposure Alters miRNA Expression Profiles in Human Lung Cells. Environ. Health Perspect..

[49] Song M.-K., Lee H.-S., Ryu J.-C. (2015). Integrated analysis of microRNA and mRNA expression profiles highlights aldehyde-induced inflammatory responses in cells relevant for lung toxicity. Toxicology.

[50] Park H.R., Lee S.E., Kim H., Lee J., Park Y.S. (2018). MicroRNA Expression Analysis of Human Pulmonary Fibroblasts Treated with Acrolein. BioChip J..

[51] Wang Z., Zhao Y., Smith E., Goodall G.J., Drew P.A., Brabletz T., Yang C. (2011). Reversal and Prevention of Arsenic-Induced Human Bronchial Epithelial Cell Malignant Transformation by microRNA-200b. Toxicol. Sci..

[52] Islam R., Zhao L., Zhang X., Liu L.-Z. (2023). MiR-218-5p/EGFR Signaling in Arsenic-Induced Carcinogenesis. Cancers.

[53] Yi S., Liu Y., Li X., Yuan X., Wang Y., Cai Y., Lei Y., Huang L., Zhang Z. (2020). The expression profile and bioinformatics analysis of microRNAs in human bronchial epithelial cells treated by beryllium sulfate. J. Appl. Toxicol..

[54] Liu Q., Zheng C., Shen H., Zhou Z., Lei Y. (2015). MicroRNAs-mRNAs Expression Profile and Their Potential Role in Malignant Transformation of Human Bronchial Epithelial Cells Induced by Cadmium. BioMed Res. Int..

[55] Wu C.-H., Hsiao Y.-M., Yeh K.-T., Tsou T.-C., Chen C.-Y., Wu M.-F., Ko J.-L. (2017). Upregulation of microRNA-4417 and Its Target Genes Contribute to Nickel Chloride-promoted Lung Epithelial Cell Fibrogenesis and Tumorigenesis. Sci. Rep..

[56] Zhang J., Zhou Y., Ma L., Huang S., Wang R., Gao R., Wu Y., Shi H., Zhang J. (2013). The Alteration of MiR-222 and Its Target Genes in Nickel-Induced Tumor. Biol. Trace Elem. Res..

[57] Solleti S.K., Bhattacharya S., Ahmad A., Wang Q., Mereness J., Rangasamy T., Mariani T.J. (2017). MicroRNA expression profiling defines the impact of electronic cigarettes on human airway epithelial cells. Sci. Rep..

[58] Tastsoglou S., Skoufos G., Miliotis M., Karagkouni D., Koutsoukos I., Karavangeli A., Kardaras F.S., Hatzigeorgiou A.G. (2023). DIANA-miRPath v4.0: Expanding target-based miRNA functional analysis in cell-type and tissue contexts. Nucleic Acids Res..

[59] Zhang Y.-K., Zhu W.-Y., He J.-Y., Chen D.-D., Huang Y.-Y., Le H.-B., Liu X.-G. (2012). miRNAs expression profiling to distinguish lung squamous-cell carcinoma from adenocarcinoma subtypes. J. Cancer Res. Clin. Oncol..

[60] A Vucic E., Thu K.L., A Pikor L., Enfield K.S., Yee J., English J.C., E MacAulay C., Lam S., Jurisica I., Lam W.L. (2014). Smoking status impacts microRNA mediated prognosis and lung adenocarcinoma biology. BMC Cancer.

